# The Rational Design of a Single‐Component Photocatalyst for Gas‐Phase CO_2_ Reduction Using Both UV and Visible Light

**DOI:** 10.1002/advs.201400013

**Published:** 2014-12-10

**Authors:** Laura B. Hoch, Thomas E. Wood, Paul G. O'Brien, Kristine Liao, Laura M. Reyes, Charles A. Mims, Geoffrey A. Ozin

**Affiliations:** ^1^ Materials Chemistry Research GroupDepartment of ChemistryUniversity of Toronto80 St. George StreetTorontoOntarioM5S 3H6Canada; ^2^ Department of Chemical Engineering and Applied ChemistryUniversity of Toronto200 College St.TorontoM5S 3E5Canada

**Keywords:** solar fuels, photocatalysis, indium oxide nanoparticles, carbon dioxide, isotope tracing

## Abstract

**The solar‐to‐chemical energy conversion of greenhouse gas CO_2_ into carbon‐based fuels** is a very important research challenge, with implications for both climate change and energy security. Herein, the key attributes of hydroxides and oxygen vacancies are experimentally identified in non‐stoichiometric indium oxide nanoparticles, In_2_O_3‐x_(OH)_y_, that function in concert to reduce CO_2_ to CO under simulated solar irradiation.

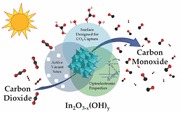

## 1 Introduction

The emerging field of solar fuels centers on storing radiant solar energy in the form of chemicals that can be used as an alternative to fossil fuels. A major goal in this field is to realize an “artificial leaf” – a material that converts light energy in the form of solar photons into chemical energy – using CO_2_ as a feedstock to generate useful chemical species. Enabling this technology will allow the greenhouse gas, CO_2_, emitted from energy production and manufacturing exhaust streams to be converted into valuable products (such as solar fuels or chemical feedstocks), thereby creating huge economic and environmental benefits by simultaneously addressing energy security and climate change issues.^1^, ^2^, ^3^, ^4^ While the global research effort with respect to the artificial leaf has focused on H_2_O splitting, the photocatalytic reduction of CO_2_ remains a significant challenge and thus form the focus of our work.^5^ This artificial leaf can exist in multiple configurations, of which gas‐phase photocatalysis has been identified as the most practical and economically feasible option for large‐scale CO_2_ reduction.^5^ Thus, the envisioned artificial leaf will be a multi‐component system that intakes large quantities of gaseous CO_2_ and pipes out large volumes of carbon‐based fuels. Clearly, the key component of this artificial leaf system is a functional material that utilizes the energy from absorbed solar photons to drive the complex multi‐electron and proton transfer reactions involved in reducing CO_2_ to fuels. As a result, there is growing interest in synthesizing semiconductor nanomaterials, which have the surface, optical, and electronic properties that can enable photocatalytic reduction of gas‐phase CO_2_ to generate solar fuels.^5^, ^6^, ^7^, ^8^, ^9^, ^10^, ^11^, ^12^ However, despite the growing interest and investment in the field, there are few examples of successful gas‐phase photocatalysts – particularly those active in the visible region of the solar spectrum – suggesting that new approaches to materials discovery are necessary.^13^

A class of materials capable of photocatalytically reducing CO_2_ are oxygen deficient metal oxides. Oxygen vacancies can function as active catalytic sites and enhance both the absorption of visible light and the photocatalytic activity of the material.^14^, ^15^ The most notable example of this is black titania, TiO_2‐x_H_x_, which exhibits a substantial increase in light absorption and photoactivity for water splitting after hydrogen treatment.^16^, ^17^ Another effective approach to increasing the photocatalytic activity of metal oxide nanomaterials is to improve the CO_2_ capture capacity of the nanoparticle surface. Several groups have demonstrated that surface hydroxides can enhance the affinity of CO_2_ for a photocatalytic surface, which can have a significant effect on the photocatalytic activity and CO_2_ reduction rates.^18^, ^19^, ^20^ Clearly, the surface, optical, and electronic properties of metal oxide nanoparticles must work in concert for photocatalytic reduction of CO_2_ to occur; understanding this relationship is critical for the advancement towards a practical global scale solar fuels technology.^13^, ^17^, ^19^, ^21^, ^22^, ^23^

Indium oxide is a material with surface, optical, and electronic properties that make it a compelling choice as a CO_2_ reduction photocatalyst. For example, its conduction band (CB) and valence band (VB) positions on an energy band diagram straddle the H_2_O oxidation and CO_2_ reduction half reaction energies required to drive artificial photosynthetic production of hydrocarbons and carbon monoxide.^4^, ^24^ Furthermore, In_2_O_3_ has a direct “forbidden” band gap where the lowest‐energy optical transition from the top of its VB to the bottom of its CB and vice‐versa is forbidden by symmetry.^25^ This “forbidden” transition has been shown in other materials to provide a built in mechanism for decreasing photo‐excited electron‐hole pair recombination rates and prolonging their lifetime, thereby greatly increasing their chances of carrying out useful surface chemistry.^26^ In addition to these beneficial optical and electronic properties, the surface properties of In_2_O_3_ have garnered interest in the field of thermally driven heterogeneous catalysis. Sun et al. have demonstrated the high activity of In_2_O_3_ towards the reverse water gas shift (RWGS) reaction at high temperatures, specifically citing CO_2_ capture as a key factor in enhancing the activity.^27^ Ye et al. have suggested from computational modeling that surface oxygen vacancies could act as active sites to promote thermally driven methanol synthesis.^28^

In this paper, hydroxylated indium oxide nanoparticles (In_2_O_3‐x_(OH)_y_), populated with surface hydroxides and oxygen vacancies, are investigated as a gas‐phase CO_2_ reduction photocatalyst. We use a temperature‐programmed thermal dehydration reaction to make In_2_O_3‐x_(OH)_y_ nanoparticles from In(OH)_3_. This simple and “green” fabrication method has numerous advantages including high atom economy, ease of scale‐up, and negligible residual carbon contamination, which can block active sites and lower the overall gas‐phase adsorption capacity and catalytic activity.^29^ Moreover, since it has been reported that the sample calcination temperature has an effect on the incident photon‐to‐electron conversion efficiency (IPCE) of In_2_O_3_ films for photoelectrochemical water splitting^30^ as well as the photocatalytic degradation of dyes,^31^ we produced, characterized and evaluated the photocatalytic performance of In_2_O_3‐x_(OH)_y_ nanoparticles prepared via thermal dehydration reactions at 250 °C, 350 °C, and 450 °C, in addition to crystalline In(OH)_3_ nanoparticles prepared from the same precursor.

Although minimal amounts of organics are present in our synthesis, we still took precaution by using ^13^C‐labelled CO_2_ (^13^CO_2_) as a reactant while testing the photocatalytic performance of these nanoparticles for CO_2_ photocatalytic activity. Light‐driven CO_2_ conversion rates reported in the literature are often low and the ubiquitous carbon contamination from carbon‐containing precursors, organic solvents, and organic additives that are used to control the size and morphology of the nanostructure can create false positive results, calling into question the validity of previously reported photoactivity.^29^ In fact, until recently few studies provided this type of evidence to support their claims, however this practice is becoming increasingly more common due to increased recognition of the importance of these tests.^32^

The overall reaction between CO_2_ and H_2_O is highly endergonic, with the majority of the energy consumed in splitting water; but vast improvements in H_2_O splitting systems have opened the possibilities for a sustainable and economically competitive supply of H_2_ as a reactant. Reactions with H_2_ and CO_2_ are thermodynamically favourable relative to those between H_2_O and CO_2_. Thus, H_2_ generated separately via solar‐driven water splitting can be used in the subsequent photocatalytic reduction of CO_2_ – such as the one described herein – to maximize the potential of the harvested sunlight. Therefore, CO_2_ reduction photocatalyts that operate in a H_2_ environment at reasonably elevated temperatures provide valuable insights into CO_2_ reduction mechanisms and increase the opportunity for researchers to discover components for a scalable artificial leaf.

The majority of CO_2_ reduction photocatalysts reported in the literature operate at room temperature or 80 °C for gas‐ and aqueous‐phase reactions, respectively.^13^, ^17^ A key insight presented in this study is that although a photocatalyst may show little or no activity at these low temperatures, by slightly elevating the reaction temperature the material can be activated and function as a CO_2_ reduction photocatalyst. These moderate temperatures can easily be reached by using simple solar trough concentrators,^33^ meaning that no external energy input is required to heat the samples.

In this work we report gas‐phase photocatalytic conversion of ^13^CO_2_ in the presence of H_2_ to generate ^13^CO at a rate as high as 0.25 μmol g_cat_^−1^ h^−1^ in a batch reactor at 150 °C under simulated solar illumination intensities of 2200 W m^−2^ on hydroxylated indium oxide nanoparticle films. We then perform the isotope tracing experiments with coupled gas chromatography‐mass spectroscopy (GC‐MS) analysis to confirm – with complete certainty – that the observed gaseous products originate from ^13^CO_2_ feedstock rather than adventitious carbon sources.^34^ Furthermore, under only visible light irradiation (λ > 420 nm) we find that our indium oxide nanoparticles photocatalytic reduce ^13^CO_2_ at a rate of 70 nmol g_cat_^−1^ h^−1^ at the same light intensity. Finally, by using a tubular flow reactor under similar conditions but with flowing CO_2_ and H_2_, the observed CO production rate can be further increased to 15 μmol g_cat_^−1^ h^−1^. Our results show that by combining the favourable optical and electronic properties inherent to indium oxide with a judiciously tailored surface, In_2_O_3‐x_(OH)_y_ nanoparticles can function as an active photocatalyst for gas‐phase CO_2_ reduction. This study provides valuable insight about key parameters for the composition selection, materials design and performance optimization of photocatalysts suited for large‐scale solar fuels production.

## 2 Results and Discussion

### 2.1 Characterization of Hydroxylated In_2_O_3‐x_(OH)_y_ via Temperature‐Controlled Decomposition of In(OH)_3_

**Figure 1:**
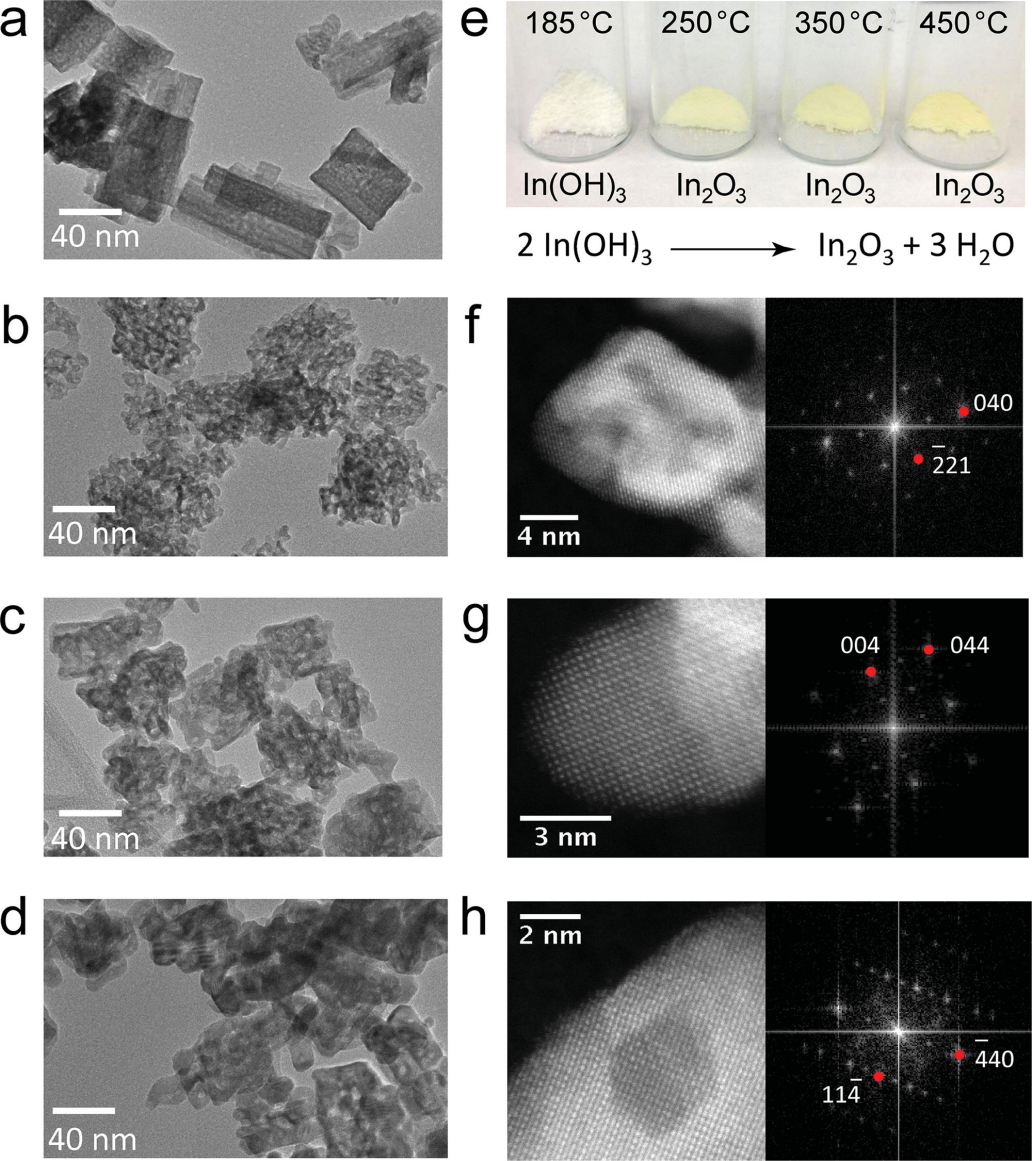
Morphology, structure, and appearance of In_2_O_3‐x_(OH)_y_ and In(OH)_3_ samples. a–d) TEM micrograph of samples treated at 185 °C, 250 °C, 350 °C, and 450 °C, respectively. All scale bars are 40 nm. e) Photographs of each sample after calcination as well as the overall reaction scheme. f–h) HRTEM‐SAADF micrographs (left) and FFTs (right) of the In_2_O_3_ samples treated at 250 °C, 350 °C and 450 °C, respectively. The zone axes indicated are (1,2,0), (1,0,0) and (2,2,1), respectively.

**Figure 2:**
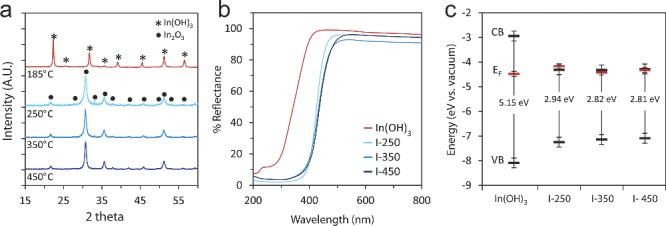
Physical characterization of In_2_O_3‐x_(OH)_y_ and In(OH)_3_ samples. a) Powder X‐ray diffraction patterns for each sample after calcination at the temperature indicated. b) Diffuse reflectance spectra of the as‐prepared nanoparticle films used in the photocatalysis experiments. c) Band position diagram of each sample. The valence band (VB) and Fermi energy (E_F_) were measured by XPS and the band gap was calculated by fitting the reflectance spectra using K‐M theory (see SI for more details on the fitting).

Hydroxylated indium oxide nanoparticles were produced via a thermal dehydration of In(OH)_3_ (**Figure** **1**). As the transition from In(OH)_3_ to In_2_O_3‐x_(OH)_y_ does not occur until approximately 210 °C, only samples heated above this temperature undergo dehydration to form indium oxide.^35^, ^36^ The transmission electron microscopy (TEM) images in Figure 1 illustrate the change in nanostructure morphology with increasing calcination temperature. The In(OH)_3_ sample calcined at 185 °C (Figure 1a) consists of large porous sheet‐like structures. As the calcination temperature is increased to 250 °C, the sheet‐like structures decompose into clusters of fused nanoparticles approximately 5 nm in diameter (Figure 1b and 1f). The overall clusters are similar in size to the In(OH)_3_ sheets, indicating that the observed porosity is likely a result of water molecules being released from the lattice as the In(OH)_3_ decomposes. As the calcination temperature increases further to 350 °C (Figure 1c and 1g) and 450 °C (Figure 1d and 1h), the average particle size increases and overall porosity of the clusters decreases. The high angle angular dark field (HAADF) high‐resolution TEM (HR‐TEM) images and the powder X‐ray diffraction patterns in **Figure** **2**a confirm that each sample consists of a single pure crystalline phase. The sample treated at 185 °C crystallizes to form pure cubic In(OH)_3_, while all other samples form pure bixbyite In_2_O_3_ with no observable In(OH)_3_ crystalline phases. For clarity, the series of In_2_O_3_ samples prepared at the different calcination temperatures of 250 °C, 350 °C, and 450 °C will be referred to as I‐250, I‐350, and I‐450 respectively.

The optical properties of each sample were determined from the diffuse reflectance spectra shown in Figure 2b. As expected, the absorption edge of In_2_O_3‐x_(OH)_y_ is significantly red‐shifted in comparison to In(OH)_3_. These diffuse reflectance spectra were fitted with a modified Kubelka‐Munk function^37^ (Figure S1, Supporting Information) to determine the optical band gap of each sample, as indicated in Figure 2c. By correlating these values with the valence band maxima and Fermi energy (E_F_) data obtained from X‐ray photoelectron spectroscopy (XPS), we can calculate the band alignment relative to the vacuum level (Figure 2c), which corresponds well to what has been reported in the literature.^25^, ^38^ The position of the Fermi energy (E_F_) just below the conduction band indicates that the as‐prepared In_2_O_3‐x_(OH)_y_ samples are n‐type semiconductors^25^, ^39^ and the overall band alignment suggests that all samples may have sufficient reducing power to photocatalytically drive gas‐phase CO_2_ reduction reactions.^13^

### 2.2 Demonstration of Photocatalytic Activity using ^13^CO_2_ Labeling

**Figure 3:**
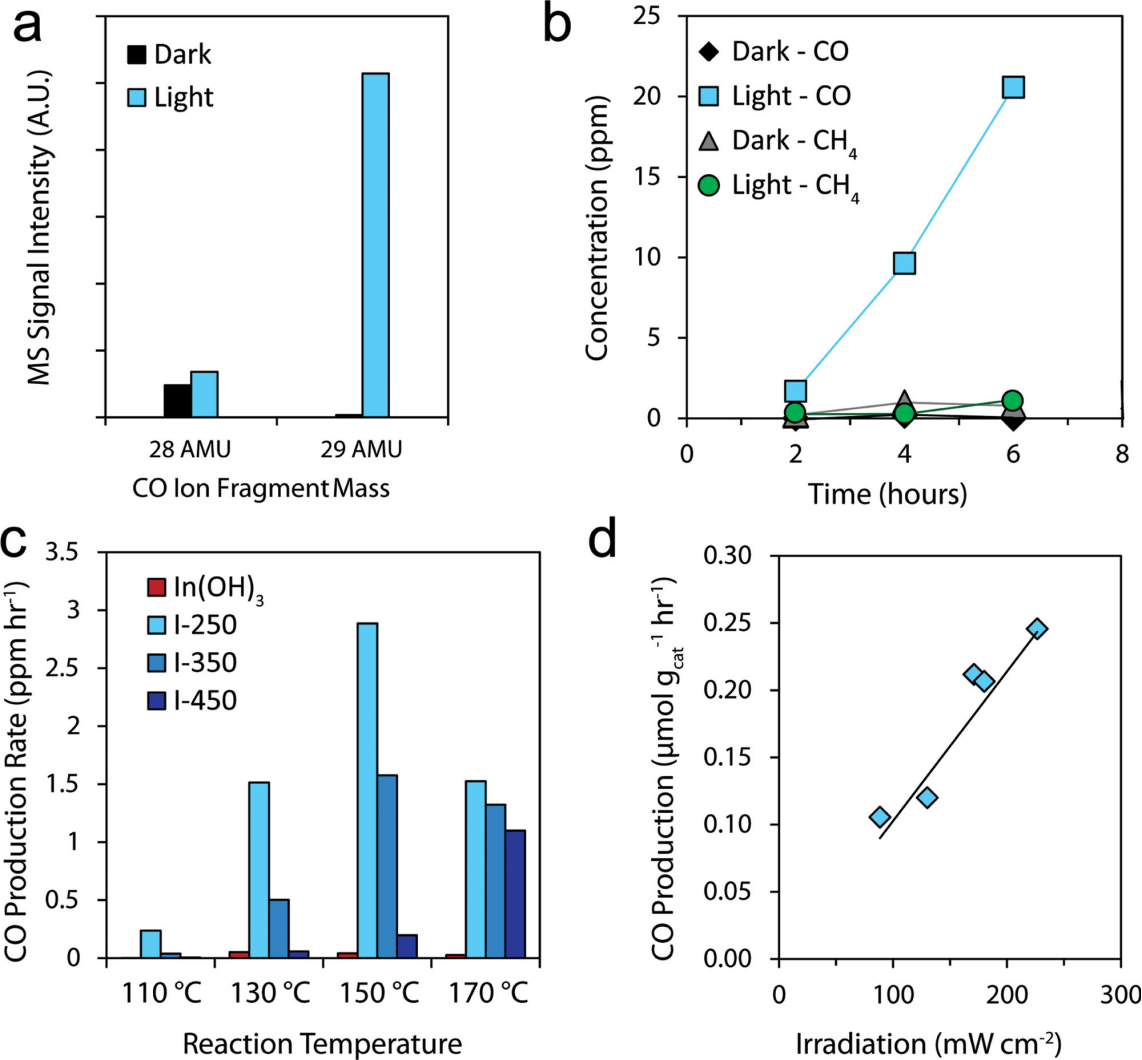
Photocatalytic performance of In_2_O_3‐x_(OH)_y_ and In(OH)_3_ samples. a) Mass spectrometry fragmentation pattern of CO generated with and without irradiation. The 28 AMU mass fragment peak corresponds to ^12^CO and the 29 AMU mass fragment peak corresponds to ^13^CO. b) Time dependent product gas formation with and without irradiation for I‐250 at a reaction temperature of 150 °C. c) Reaction temperature dependence of CO production rates for In(OH)_3_, I‐250, I‐350 and I‐450 measured under 1 sun irradiation. d) Effect of light intensity on the CO production rate of I‐250 under irradiation from a 500 W Xe lamp at different light intensities.

In order to confirm the photocatalytic activity of the In_2_O_3‐x_(OH)_y_ samples, carbon‐13 labelled carbon dioxide (^13^CO_2_) is used as a tracer molecule to identify products from the photocatalytic reaction in the presence or absence of irradiation. This is an important step that determines whether the carbon source of the products originates from CO_2_ or from adventitious carbon contamination on the sample.^29^ After 16 hours of reaction at 150 °C under both light (800 W m^−2^ using a 1000 W metal halide bulb) and dark conditions, I‐250 produced CH_4_, CO, H_2_O and trace amounts of higher chain hydrocarbons. It was found that CH_4_ is produced at an average rate of 11 nmol g_cat_^−1^ hour^−1^ under irradiation, and was produced even in the absence of irradiation. It was also observed that the CH_4_ production rate decreased with subsequent batch reactions. The product ion‐fragmentation pattern obtained using GC‐MS (Figure S2, Supporting Information) shows that the intensity of the 16 AMU parent peak of ^12^CH_4_ is significant, while the intensity of the 17 AMU parent peak of ^13^CH_4_ is barely above noise level. This suggests CH_4_ is produced by the decomposition or reaction of adventitious carbon on the surface and not from the CO_2_ feedstock. In contrast, it was found that CO is unequivocally a product of CO_2_ photocatalytic activity and is produced only under light irradiation at an average rate of 0.25 μmol g_cat_^−1^ hour^−1^ in batch reactors. **Figure** **3**a shows the relative intensities of the 28 AMU parent peak of ^12^CO and the 29 AMU parent peak of ^13^CO under both dark and light conditions. The absence of the 29 AMU peak in the dark and the significant increase in its intensity under irradiation demonstrates that the conversion of ^13^CO_2_ to ^13^CO is light driven. This finding is further confirmed by a comparison of time dependent product formation. Figure 3b shows that CO production increases linearly with time only under irradiation, while CH_4_ production remains at near baseline levels under both dark and light conditions.

### 2.3 Investigating the Effects of Sample Calcination Temperature, and Reaction Temperature on Photocatalytic Activity

Previous studies have indicated that sample calcination temperature can have a strong effect on the aqueous‐phase photoelectrochemical performance of indium oxide nanostructures.^30^, ^31^ In order to determine if the calcination temperature also affects the photocatalytic performance in the gas‐phase, we measured the CO production rates under light and dark conditions for a set of In_2_O_3‐x_(OH)_y_ sample films, each loaded with 20 mg of sample and calcined at different temperatures (I‐250, I‐350 and I‐450) as well as a 20 mg In(OH)_3_ control sample film. The CO production rates for these samples under 80 mW cm^−1^ of irradiation using a 1000 W metal halide lamp at a range of reaction temperatures are shown for each sample in Figure 3c. In general, indium oxide samples calcined at lower temperatures produced CO at higher rates for the range of reaction temperatures studied. The photocatalytic CO_2_ reduction rate at 150 °C for I‐250 is roughly twice that of I‐350 and approximately an order of magnitude greater than that of I‐450. The In(OH)_3_ control produced almost no CO at any temperature. Both I‐250 and I‐350 demonstrate an increase in photocatalytic CO production with increasing reaction temperature, reaching a maximum at 150 °C and decreasing in activity at 170 °C. Sample I‐450, on the other hand, shows an increase in reaction activity at 170 °C relative to 150 °C, in contrast to I‐250 and I‐350. Due to limitations on the maximum operating temperature of the photoreactors, the reaction was not investigated at temperatures higher than 170 °C. Alternative reactor designs are currently being investigated to understand these trends at higher temperatures. The effect of irradiation on CO production rates for each sample at different reaction temperatures is also shown in Figure S3, Supporting Information. As expected, all samples produced very little CO under dark conditions, with a maximum of 2.8 nmol g_cat_^−1^ h^−1^ measured at 170 °C for I‐250, demonstrating that the observed gas phase CO_2_ reduction is a light‐driven process.

### 2.4 Investigating the Effect of Light Intensity and Spectral Distribution on Photocatalytic Activity

In order to determine the effect of light intensity on the CO production rate, a 20 mg I‐250 film was irradiated with a Newport 300 W Xe Lamp fitted with an AM1.5 filter to simulate the solar spectrum. The photocatalytic activity of the sample was tested at 150 °C under varying light intensities from 0.8 to 2.2 suns. Figure 3d shows a linear increase in CO production rate with increasing light intensity, which further confirms that the CO_2_ to CO conversion is a light‐driven reaction. A single sample was used for the duration of these measurements, demonstrating the robustness of this photocatalyst.

The spectral dependence of CO production was also investigated (Figure S4, Supporting Information). A single 20 mg I‐250 film was irradiated with a Newport 300W Xe Lamp, fitted with either an AM1.5 filter or an AM1.5 filter combined with either a 420 nm or a 615 nm high‐pass filter. The light intensity was set to 1700 W m^−2^, using a focusing lens to adjust the intensity and a calibrated reference cell to measure the output. When the I‐250 sample was initially irradiated with the AM1.5 filtered light, a CO production rate of 0.20 μmol g_cat_^−1^ hour^−1^ was observed. The second run – with the additional 420 nm high pass filter that cut off all wavelengths with energy greater than 420 nm – produced CO at a rate of 70 nmol g_cat_^−1^ hour^−1^. No CO was detected when a 615 nm high‐pass filter was used. Finally, a repeat of the initial measurement using only the AM1.5 filter was conducted, reproducing the rate of 0.20 μmol g_cat_^−1^ hour^−1^. These results demonstrate that not only is the I‐250 capable of converting gaseous CO_2_ to CO using only visible light, which correlates well with the diffuse reflectance measurements, but also that In_2_O_3‐x_(OH)_y_ is stable under these reaction conditions and can produce rates consistent with the initially measured values even after being irradiated continuously for 4 days.

### 2.5 Photocatalytic CO_2_ Reduction Rates using a Flow Reactor

In an attempt to simulate more industrially relevant conditions, preliminary photocatalytic rate measurements were carried out in a tubular fixed bed flow reactor irradiated with a Newport 300W Xe lamp. This investigation of the In_2_O_3‐x_(OH)_y_ nanoparticles revealed that under similar conditions to those in the batch photoreactors (2200 mW cm^−2^, 150 °C and 3 atm), under flowing CO_2_ and H_2_, CO is photocatalytically produced at a rate of 15 μmol g_cat_^−1^ h^−1^ with 24 mg (3 cm bed length) of the I‐250 nanoparticle powder sample. These rates are higher than previously reported CO production rates for other single component metal oxides; for instance 1.61 μmol g_cat_^−1^ h^−1^ for MgO,^40^ and 0.56 μmol g_cat_^−1^ h^−1^ for ZrO_2_.^41^ This increased activity is the focus of a current on‐going investigation. Additionally, in order to ensure consistency in this study, the photocatalytic rate data presented here are limited to those from identical batch photoreactors. This mitigates effects caused by variations in particle size between I‐250, I‐350, and I‐450 and allows us to more accurately make comparisons between these samples. Differences in particle size of powders packed in a catalyst bed can result in substantial variations in pressure gradients, making comparisons between samples more difficult.

### 2.6 Characterization of Surface Hydroxides and Oxygen Vacancies

**Figure 4:**
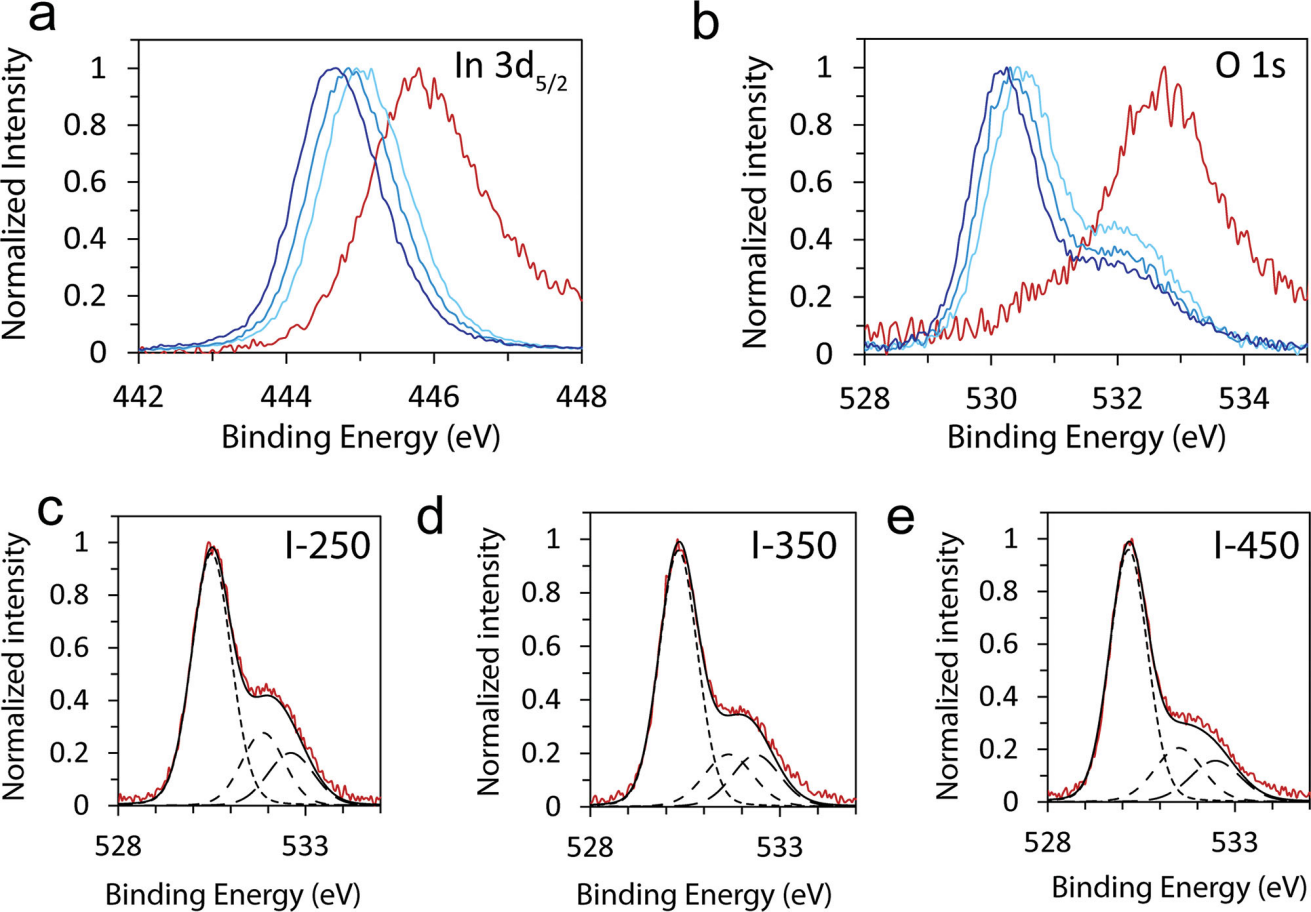
Comparison of XPS spectra for In_2_O_3‐x_(OH)_y_ and In(OH)_3_ samples. XPS spectra of the a) In3d_5/2_ and b) O1s peaks. c–e) De‐convolution of the high‐resolution O1s spectra for the I‐250, I‐350 and I‐450 samples. The main peak at 530.3 eV is attributed to indium oxide. The two additional shoulder peaks at 531.7 eV and 532.5 eV are attributed to oxygen vacancies in the structure and surface OH groups, respectively.

In order to further understand the effects of calcination temperature on the photocatalytic activity, XPS measurements were conducted. **Figure** **4**a shows that the In3d_5/2_ core level peak shifts to a lower binding energy as the calcination temperature is increased, indicating an increase in charge density around the In atoms as a result of the removal of OH groups. The O1s core level spectra in Figure 4b shows a sharp contrast between In(OH)_3_ and In_2_O_3‐x_(OH)_y_ samples. There is an approximately 2.5 eV shift to lower binding energy of the main O1s peak, from 532.7 eV for In(OH)_3_ to 530.2 eV for I‐450. Additionally, a shoulder peak appears in the O1s core level peak of the In_2_O_3‐x_(OH)_y_ samples, indicating that there is more than one chemical state of oxygen present in the structure. Indeed, the O1s peak for the In_2_O_3‐x_(OH)_y_ samples can be de‐convoluted into three distinct peaks: the main oxide peak at 530.3 eV and two additional peaks at 531.7 eV and 532.5 eV (Figure 4c–e). The peak at 531.7 eV is commonly attributed to the presence of oxygen vacancies in the structure.^42^, ^43^ It is also consistent with the observed n‐type position of the Fermi‐levels relative to the conduction bands (Figure 2c), which is typically a result of non‐stoichiometry.^44^ This vacancy peak is shifted to a higher binding energy relative to the main oxide peak. This is a result of the change in O interaction with an In centre that is more reduced in character because it is surrounded by less than six O atoms (due to the oxygen vacancies). The peak at 532.5 eV has been attributed to surface OH groups^42^ and agrees well with the O1s spectra for the pure In(OH)_3_ peak. From these plots in Figure 4c–e, it is clear that the shoulder peak – with contributions from both vacancies and surface hydroxides – decreases with increasing calcination temperature. XPS measurements were also conducted on samples exposed to reaction conditions and results indicate a slight change in the shoulder peak of the O1s spectra. Current on‐going research aims to investigate this material in situ to determine how the surface may change during reaction.

The hydroxide content of the samples was investigated further by both Fourier transform infrared (FT‐IR) spectroscopy and thermogravimetric measurements. The intensity of the OH stretches in the FT‐IR spectra (Figure S5, Supporting Information) decreases with increasing calcination temperature, illustrating that samples treated at higher temperatures have lower hydroxide contents. In order to establish the extent of hydroxide loss during synthesis, each In_2_O_3‐x_(OH)_y_ sample was synthesized in situ within a thermogravimetric analyzer. The weight loss observed was attributed to hydroxide condensation to form bridging oxides. Figure S6, Supporting Information, shows the weight loss of each sample after calcination under air flow for 3 hours. It is clear that lower calcination temperatures correspond to less overall weight loss and result in stabilization at a higher relative weight. By comparing these plots to the theoretical maximum weight loss (when all hydroxides are converted to bridging oxides), it is apparent that I‐450 should have almost no hydroxides left. I‐250 and I‐350, on the other hand, have additional weight above the theoretical value, which we attribute to the retained hydroxyl groups. From this data and the Brunauer‐Emmett‐Teller (BET) surface area described below, we estimate that the surface hydroxide coverage is on the order of 6 μmol m^−2^ for I‐250 and 3 μmol m^−2^ for I‐350. It is expected that the combination of both hydroxide groups and oxygen vacancies is a key feature of our functioning In_2_O_3‐x_(OH)_y_ nanoparticle photocatalysts and that both of these entities are present and work in concert at active sites.

### 2.7 Correlation of CO_2_ Capture Capacity with Observed Photocatalytic Rates

**Figure 5:**
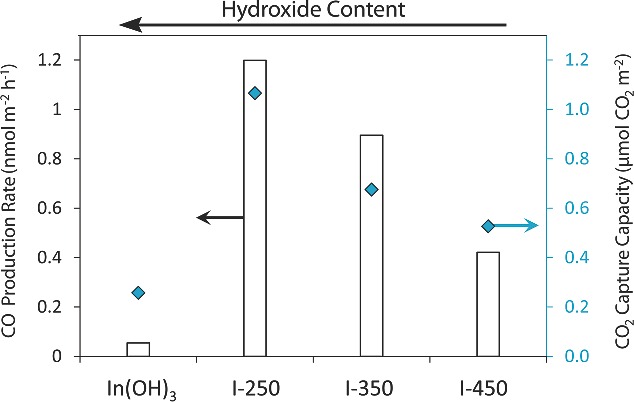
Comparison between the CO_2_ reduction rates and the CO_2_ capture capacity of In_2_O_3‐x_(OH)_y_ for samples prepared at different calcination temperatures. The white bars correspond to the CO production rates at 150 °C and the diamonds correspond to the CO_2_ capture capacity of each sample at 150 °C.

To clarify the observed trend in CO production rates between In_2_O_3‐x_(OH)_y_ samples, the CO_2_ capture capacity was determined for each sample at 150 °C, the reaction temperature at which the highest CO production rates for all samples were observed. Furthermore, in order to more accurately compare the CO_2_ capture capacity and the photoactivity, both are normalized to the surface area of each sample, determined using the BET method. The surface areas for the In(OH)_3_, I‐250, and I‐350 were remarkably similar at 124.7 m^2^ g^−1^, 125.0 m^2^ g^−1^, and 129.6 m^2^ g^−1^, respectively. Only I‐450 had a significantly lower surface area at 90.0 m^2^ g^−1^, which is likely a result of some nanoparticle sintering at the higher calcination temperatures. **Figure** **5** shows the surface‐area‐normalized CO_2_ capture capacities for each sample plotted together with their corresponding CO production rates. There is a notable strong correlation between CO_2_ reduction rates and the normalized CO_2_ capture capacity.

### 2.8 Designing a Surface for CO_2_ Photocatalytic Activity

The affinity of a photocatalyst's surface for CO_2_ has been identified in this study, as well as in others,^18^, ^19^, ^20^ as a critical factor influencing photocatalytic activity. As Figure 5 demonstrates, the CO_2_ capture capacity of the In_2_O_3‐x_(OH)_y_ nanoparticles corresponds very well with photo‐reactivity, indicating that CO_2_ adsorption plays an important role in the light‐driven reaction. Intuitively, CO_2_ molecules must be able to approach and interact with the surface for a sufficient amount of time in order for electron transfer to occur. Surface hydroxides have a known affinity for the Lewis‐acidic CO_2_.^45^ This could explain the strong positive correlation between CO_2_ capture capacity and hydroxide content. However, while the In(OH)_3_ control sample has the highest hydroxide content and a similar surface area to that of I‐250, it also has a significantly lower CO_2_ capture capacity. This indicates that surface hydroxides alone are not sufficient to facilitate CO_2_ capture and photocatalytic reduction of CO_2_.

In addition to hydroxides, the surface of the In_2_O_3‐x_(OH)_y_ nanoparticles is also populated with oxygen vacancies. The presence of these oxygen vacancies in the In_2_O_3‐x_(OH)_y_ samples is supported by both the de‐convolution of the XPS O1s core level peaks (Figure 4c–e) as well as the n‐type position of the Fermi‐levels relative to the conduction bands (Figure 2c), which is typically a result of non‐stoichiometry.^44^ It is apparent from both figures that I‐250 has the largest peak associated with oxygen vacancies as well as the highest Fermi energy, implying a higher abundance of vacancies compared to the other temperature‐treated In_2_O_3‐x_(OH)_y_ samples. The increase in oxygen vacancies for I‐250 may result from the natural increase in surface defect sites as the particle size decreases. Surface oxygen vacancies may also arise from the crystal structure of In_2_O_3_. The cubic In_2_O_3‐x_(OH)_y_ samples have a bixbyite structure, which can be understood as the CaF_2_‐type lattice with 25% of the tetrahedral anion sites vacant. This additional space in the bixbyite structure may result in more dynamic flexibility, especially at the nanoparticle surface, allowing for more atomic mobility in the lattice; indeed In_2_O_3_ is a known solid ionic and protonic conductor.^46^ Additionally, these intrinsic oxygen vacancies may increase the stability of the vacant surface sites, allowing the material to remain stable under reaction conditions. By contrast the stoichiometric In(OH)_3_ with its perovskite structure does not have a significant concentration of surface oxygen vacancies. The implied necessary combination of surface hydroxides and oxygen vacancies could provide an explanation for the stark difference in CO_2_ capture capacity and photocatalytic activity of In(OH)_3_ and I‐250.

Surface oxygen vacancies may also form due to interactions between surface oxygen sites and H_2_ or CO under elevated reaction temperatures. In_2_O_3_ has been investigated experimentally^47^, ^48^ and theoretically^28^, ^49^ in order to determine surface oxygen vacancy formation under reducing environments (H_2_ or CO) and oxidizing environments (O_2_, CO_2_ or H_2_O). Bielz et al. have demonstrated that oxygen vacancies are generated on the surface of In_2_O_3_ in H_2_ environments at temperatures greater than 125 °C, as shown in Equation (1):^47^

(1) $$H_{2}+{\left[O\right]}_{lattice}\Rightarrow H_{2}O+{\left[\;\;\;\;\;\;\;\;\right]}_{lattice}$$

H_2_ reduction of the surface under these reaction conditions may suggest the temperature dependence of the CO production rates for the In_2_O_3‐x_(OH)_y_ is due to the availability of surface oxygen vacancies (Figure 3c). As shown, very little CO is observed at 110 °C (sample I‐250 is the only sample to produce a significant amount of CO at 110 °C). However, at reaction temperatures above 130 °C, CO production under light irradiation is significant. While the photocatalytic production increases from 110 °C to 150 °C, the activity decreases at 170 °C. The decrease in CO production at 170 °C may be due to oxidation of CO by lattice oxygen on the In_2_O_3‐x_(OH)_y_ surface as shown in Equation (2).^48^

(2) $$CO+{\left[O\right]}_{lattice}\Rightarrow CO_{2}{\left[\;\;\;\;\;\;\;\;\;\;\right]}_{lattice}$$

An alternative explanation for the observed dependence on temperature trend is the adsorption and desorption of molecules at the surface. At higher temperatures, product molecules such as H_2_O, which can block active sites, may desorb enabling more turnovers at these active sites.^50^ Since it is observed that In_2_O_3‐x_OH_y_ samples achieve a maximum efficiency at 150 °C, this may indicate that 150 °C is a “sweet spot,” combining efficient CO_2_ adsorption and efficient CO and H_2_O desorption.

It is also possible that the reaction takes place between CO_2_ and the surface oxygen vacancies, as outlined in Equation (1) to produce CO through a surface oxidation reaction. However, pre‐reducing the sample in H_2_ at elevated temperature followed by a batch reaction in CO_2_ yielded no trace of CO under irradiation. Thus it is believed that the observed reaction is the reverse water gas shift (RWGS) reaction as shown in Equation (3).

(3) $$CO_{2}+H_{2}\Rightarrow CO+H_{2}O$$

While we have observed water as a product, an exact reaction stoichiometry is difficult to quantify to complete a mass balance for the proposed RWGS reaction due to uncertainties created by the strong interaction of water with the tubing connecting the reactors to the GC and GC‐MS.

## 3 Conclusion

A functional single component CO_2_ reduction photocatalyst must have surface, optical, and electronic properties working in concert for photocatalytic reduction of CO_2_ to occur in the gas phase. In this study the In_2_O_3‐x_(OH)_y_ nanoparticles demonstrate activity for the photocatalytic reduction of CO_2_ in the presence of H_2_ at temperatures as low as 130 °C using both ultraviolet and visible light. Our work strongly suggests that the observed activity of In_2_O_3‐x_(OH)_y_ samples is associated with surface populations of oxygen vacancies and hydroxides, which may act in concert as active sites for CO_2_ adsorption and charge transfer under simulated solar irradiation.

We have produced a series of nanostructured In_2_O_3‐x_(OH)_y_ materials via a temperature controlled thermal dehydration of In(OH)_3_. Using ^13^CO_2_ as a tracer molecule, strong light and temperature‐dependent photocatalytic reduction of gaseous ^13^CO_2_ to ^13^CO is confirmed in the presence of H_2_. The surface hydroxide and oxygen vacancy content strongly correlates with both an increase in ^13^CO_2_ capture capacity and an increase in photocatalytic activity for ^13^CO production. By combining the favourable surface, electronic and optical properties of nanostructured In_2_O_3‐x_(OH)_y_ with the bixybite crystal structure and its enhanced CO_2_ capture capabilities, we have demonstrated a combination of key components to be considered in the discovery, optimization, and scaling of new and efficient gas‐phase CO_2_ reduction photocatalysts for solar fuels production.

## 4 Experimental Section

*Synthesis of In_2_O_3‐x_(OH)_y_ Nanoparticles*: An In(OH)_3_ precursor was synthesized and subsequently dehydrated into In_2_O_3_ nanoparticles following a modified version of a previously published procedure.^51^ All chemicals were used as received without any further purification. In a typical synthesis, of indium(III) chloride (3.6 g, 16.2 mmol, Sigma Aldrich, 98%) was dissolved in a 3:1 solution (72 mL) of of anhydrous ethanol (Commercial Alcohols) and deionized, nanopure water (resistivity 18.2 MΩ cm). In a separate beaker, a 3:1 mixture of ethanol and ammonium hydroxide was prepared by combining aqueous ammonium hydroxide (18 mL, Caledon, 28–30% adjusted to 25 wt% with deionized water) and of anhydrous ethanol (54 mL). The solutions were rapidly combined, resulting in the immediate formation of a white precipitate. To control the particle size, the resulting suspension was immediately immersed in a pre‐heated oil bath at 80 °C and stirred for 10 min. The suspension was then removed from the oil bath and allowed to cool to room temperature. The precipitate was separated via centrifugation and washed 3 times with deionized water. The precipitate was sonicated between washings to ensure adequate removal of any trapped impurities and then dried overnight at 80 °C in a vacuum oven. The dried precursor powder was finely ground with a mortar and pestle and calcined for 3 hours in air at 185 °C, 250 °C, 350 °C, and 450 °C. Sample films were prepared for photocatalytic testing by drop casting 20 mg of each sample powder – suspended via sonication in deionized, nanopure water (3 ml) – onto 1” × 1” binder free borosilicate glass microfiber filters (Whatman, GF/F, 0.7 μm) placed on top of a vacuum filtration funnel that was under very weak vacuum. This sample loading was selected to optimize CO production rates, as illustrated in Figure S7, Supporting Information.

*Physical Characterization*: Powder X‐ray diffraction (PXRD) was performed on a Bruker D2‐Phaser X‐ray diffractometer, using Cu Kα radiation at 30 kV. Nitrogen Brunauer‐Emmet‐Teller (BET) adsorption isotherms were obtained at 77 K using a Quantachrome Autosorb‐1‐C. Sample morphology was determined using a JEOL‐2010 high resolution transmission electron microscope (HR‐TEM). Fourier transform infrared spectroscopy (FT‐IR) was performed using a Perkin Elmer Spectrum‐One FT‐IR fitted with a universal attenuated total reflectance (ATR) sampling accessory with a diamond coated zinc selenide window. Diffuse reflectance of the samples was measured using a Lambda 1050 UV/VIS/NIR spectrometer from Perkin Elmer and an integrating sphere with a diameter of 150 mm. Sample weight loss during the calcination process was determined by placing approximately 10 mg of un‐calcined indium hydroxide precursor in a TA Instruments Q500 thermogravimetric analyzer, jumping to the set temperature of either 250 °C, 350 °C or 450 °C and holding for 3 hours under a flow of compressed air. The sample weight was determined using the built‐in ATI CAHN C‐34 microbalance. The film morphology and thickness was characterized by scanning electron microscopy using a QUANTA FEG 250 ESEM. The borosilicate glass microfiber filters were used as a substrate to provide increased surface area as well as mechanical stability. Figure S8, Supporting Information, shows SEM micrographs of a typical In_2_O_3_ sample on the filter. Figure S8a, Supporting Information, shows a cross‐section of the film, indicating its thickness is approximately 50 μm. The magnified image shown in Figure S8b, Supporting Information, indicates that the as‐prepared sample maintains its high porosity, an important factor for gas‐phase reactions.

X‐ray photoelectron spectroscopy (XPS) was performed using a Perkin Elmer Phi 5500 ESCA spectrometer in an ultrahigh vacuum chamber with base pressure of 1 × 10^−9^ Torr. The spectrometer uses an Al Kα X‐ray source operating at 15 kV and 27 A. The samples used in XPS analyses were prepared by drop‐casting aqueous dispersions onto p‐doped Si(100) wafers in the case of the In_2_O_3_ samples and fluorine‐doped tin oxide substrate in the case of the In(OH)_3_ sample. All data analyses were carried out using the Multipak fitting program and the binding energies were referenced to the NIST‐XPS database and the Handbook of X‐ray photoelectron spectroscopy.^52^, ^53^

*Gas‐Phase Photocatalytic Measurements*: Gas‐phase photocatalytic rate measurements were conducted in a custom fabricated 1.5 mL stainless steel batch reactor with a fused silica view port sealed with Viton O‐rings. The reactors were evacuated using an Alcatel dry pump prior to being purged with the reactant gases H_2_ (99.9995%) and CO_2_ (99.999%) at a flow rate of 6 mL min^–1^ and a stoichiometry of either 4:1 (stoichiometric for Sabatier reaction) or 1:1 (stoichiometric for reverse water gas shift reaction). During purging, the reactors were sealed once they had been heated to the desired temperature. The reactor temperatures were controlled by an OMEGA CN616 6‐Zone temperature controller, with a thermocouple placed in contact with the sample. The pressure inside the reactor during reaction was monitored during the reaction using an Omega PX309 pressure transducer. Reactors were irradiated with a 1000 W Hortilux Blue metal halide bulb (the spectral output is shown in Figure S9, Supporting Information) for a period of 16 hours. Product gases were analyzed by a flame ionization detector (FID) and thermal conductivity detector (TCD) installed in a SRI‐8610 Gas Chromatograph (GC) with a 3' Mole Sieve 13a and 6‘Haysep D column. Isotope tracing experiments were performed using ^13^CO_2_ (99.9 atomic% Sigma Aldrich). The reactors were evacuated prior to being injected with ^13^CO_2_, followed by H_2_. Isotope product gases were measured using an Agilent 7890A gas chromatographic mass spectrometer (GC‐MS) with a 60 m GS‐Carbonplot column fed to the mass spectrometer. The spectral dependence of the photoactivity of the In_2_O_3_ nanoparticles was investigated using a Newport 300 W Xe Lamp (the spectral output is shown in Figure S9, Supporting Information) fitted with a combination of AM1.5, 420 nm high‐pass and 615 nm highpass filters. Since each filter reduced the total irradiation intensity, the beam was focused using collimating lenses to maintain an irradiation intensity of 100 mW cm^–2^. The spectral output was measured with a StellarNet Inc spectrophotometer. Irradiation intensity was measured by a Newport 91150V calibrated reference cell and meter. Additional photocatalytic rate measurements were carried out in a borosilicate tube (3 mm outer diameter and 2.5 mm inner diameter) reactor packed with 24 mg (3 cm bed length) of In_2_O_3‐x_(OH)_y_ nanoparticle powder. Quartz wool was used to support either end. The reactor was held in a custom designed stand. Heating was supplied from a heated copper block fixed below the fixed catalyst bed. The top of the reactor was exposed in order to allow light irradiation from a Newport 300 W Xe Lamp (at a distance of 4 cm and a light intensity of 2.2 suns). The reactor was purged with H_2_ (99.9995%) and CO_2_ (99.999%) at a flow rate of 10 mL min^–1^ and a stoichiometric ratio of 1:1 (stoichiometric for reverse water gas shift reaction). The reactor temperatures were controlled by an OMEGA CN616 6‐Zone temperature controller. A thermocouple was in contact with the top of the reactor so that the reactor maintained a constant temperature of 150 °C.

*CO_2_ Capture Capacity*: The CO_2_ capture capacity of each sample was measured by thermogravimetric analysis (TGA) with a TA Instruments Q500 thermogravimetric analyzer. A desorption step was first carried out under N_2_ flow at a rate of 100 mL min^–1^ with a temperature ramp of 10 °C min^–1^ up to 150 °C; the temperature was held at 150 °C for 3 hours. To measure the amount of CO_2_ adsorption, the gas was then switched to 100% dry CO_2_ at a flow rate of 100 mL min^–1^; the temperature was then maintained at 150 °C for 10 hours. The weight gain observed during this adsorption step was used to calculate the CO_2_ capture capacity of the sample. Desorption of CO_2_ was performed by switching the gas flow back to N_2_ flow for 5 hours while keeping the temperature constant at 150 °C.

## Supporting information

As a service to our authors and readers, this journal provides supporting information supplied by the authors. Such materials are peer reviewed and may be re‐organized for online delivery, but are not copy‐edited or typeset. Technical support issues arising from supporting information (other than missing files) should be addressed to the authors.

Supplementary
