# Supplementary material for: The Rational Design of a Single‐Component Photocatalyst for Gas‐Phase CO2 Reduction Using Both UV and Visible Light
Source: Adv Sci (Weinh). 2014 Dec 10;1(1):1400013. doi: 10.1002/advs.201400013 (PMC5115262; doi:10.1002/advs.201400013)
Supplement: Supplementary file 1 — Supplementary [file ADVS-1-0c-s001.pdf]

Copyright WILEY-VCH Verlag GmbH & Co. KGaA, 69469 Weinheim, Germany, 2014.

Supporting Information

for *Adv. Sci.*, DOI: 10.1002/advs. 201400013

The Rational Design of a Single-Component Photocatalyst for Gas-Phase CO_2 Reduction Using Both UV and Visible Light

*Laura B. Hoch, Thomas E. Wood, Paul G. O'Brien, Kristine Liao, Laura M. Reyes, Charles A. Mims, and Geoffrey A. Ozin**

Supporting Information

The Rational Design of a Single-Component Photocatalyst for Gas-Phase CO_2 Reduction Using Both UV and Visible Light

*Laura B. Hoch, Thomas E. Wood, Paul G. O'Brien, Kristine Liao, Laura M. Reyes, Charles A. Mims and Geoffrey A. Ozin **

Supporting Information:

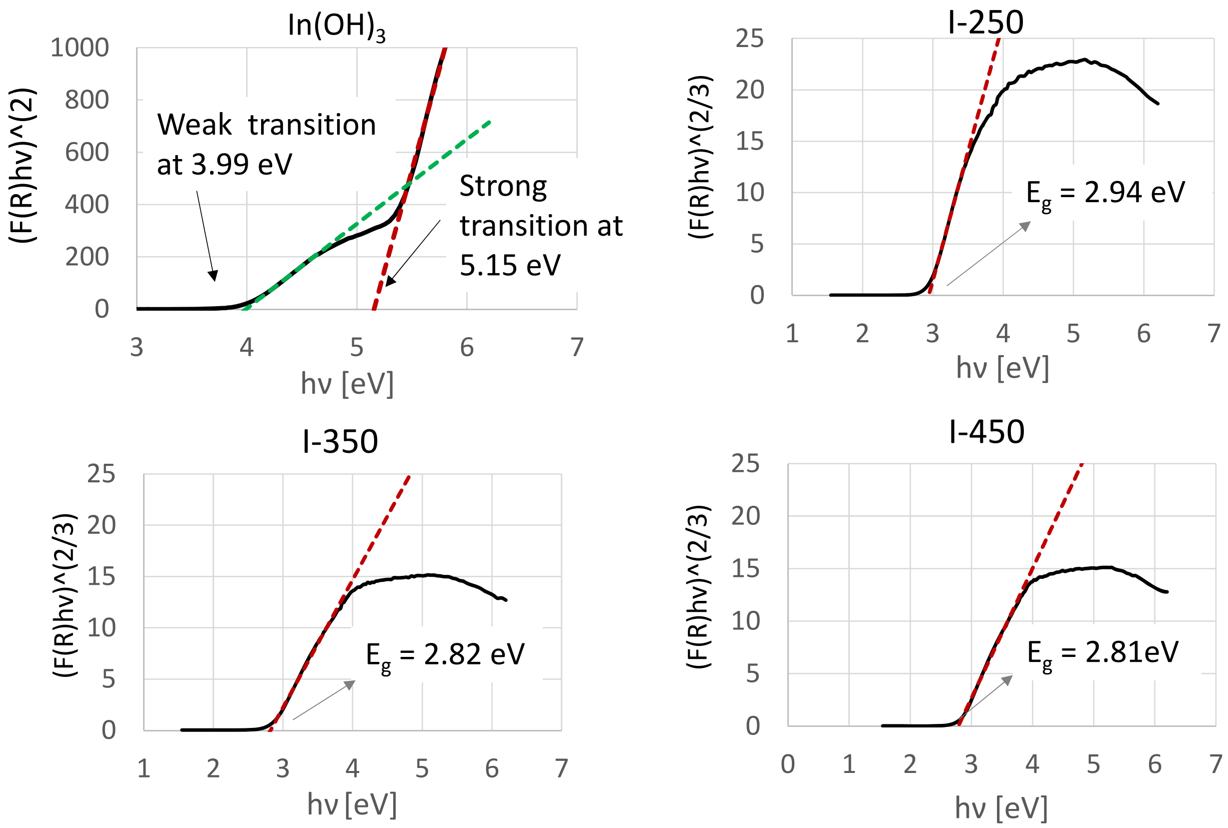

Figure S1. Estimation of the electronic band gap of the $In_2O_{3-x}(OH)_y$ and $In(OH)_3$ powders using a modified Kubelka-Munk function. Specifically, $(F(R)*h\nu)^n$ is plotted as a function of photon energy for samples (a) $In(OH)_3$, (b) I-250, (c) I-350 and (d) I-450 where $F(R) = (1-R)^2/2R$. R is the diffuse reflectance of the films loaded onto the borosilicate supports and n was set to 1/2 for the $In(OH)_3$ film and 3/2 for the $In_2O_{3-x}(OH)_y$ films. The linear portion of the plot was extrapolated and its intercept with the abscissa provided the band-gap estimate.

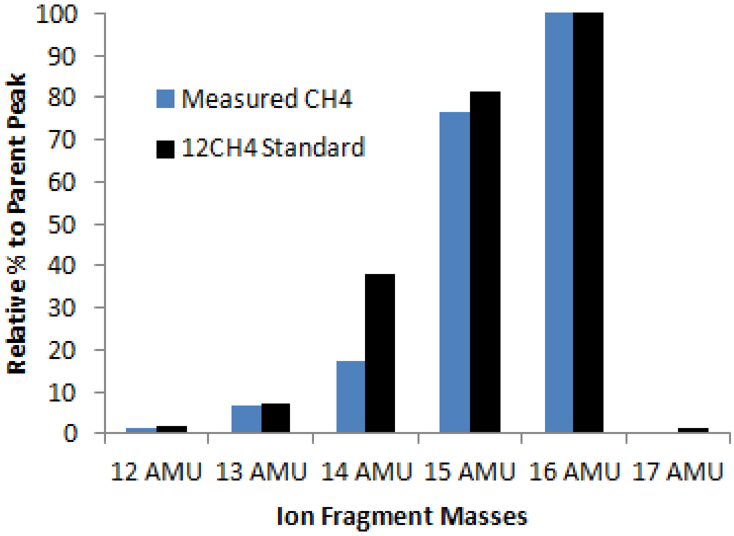

Figure S2. GC-MS fragmentation of CH_4 produced from $^{13}CO_2$ isotope tracing measurements of I-250 plotted in contrast to a $^{12}CH_4$ standard. This result confirms that methane does not originate from the carbon dioxide introduced into the reactor and is therefore likely a result of adventitious carbon contamination on the surface of the sample.

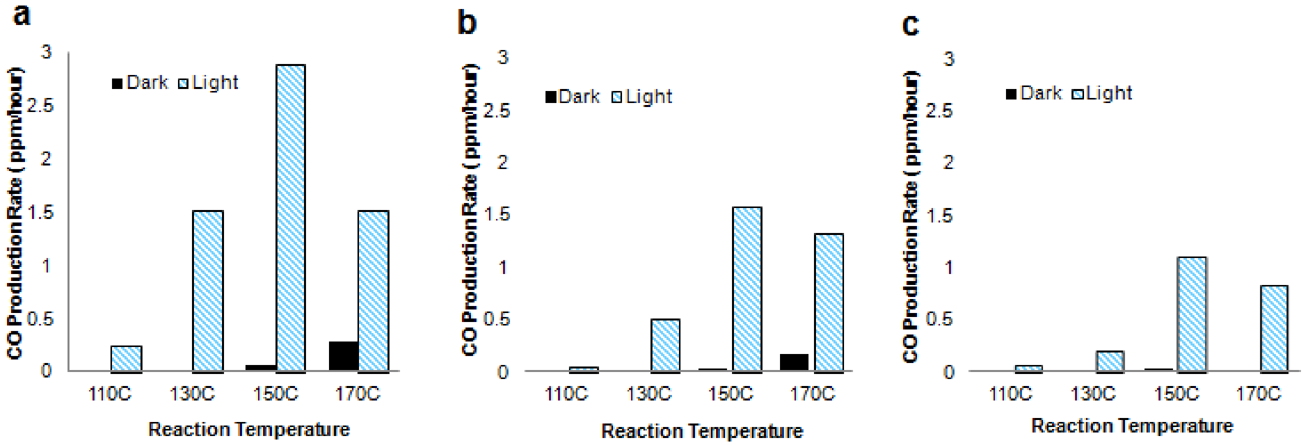

Figure S3. CO production dependence on the presence of light and reaction temperature: CO production in the presence and absence of light and at temperatures ranging from 110 °C to 170 °C measured for samples (a) I-250, (b) I-350, and (c) I-450.

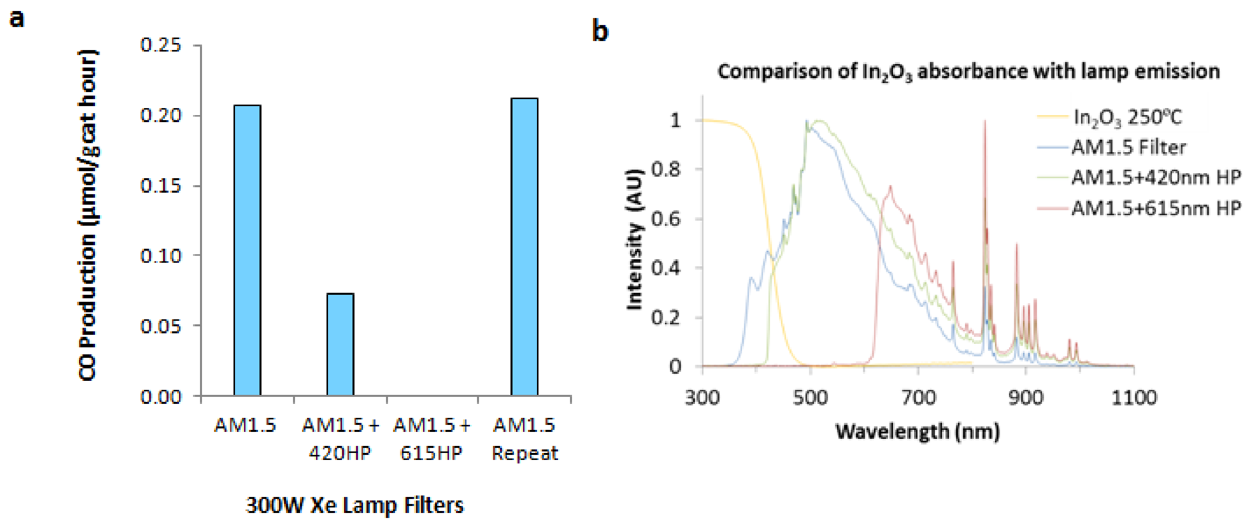

Figure S4. (a) Effect of spectral profile on CO production rate for a single I-250 film under irradiation from a Newport 300 W Xe Lamp fitted with a combination of AM1.5, 420 nm high pass, or 615 nm high pass filters. (b) Comparison of the estimated absorption spectrum of an I-250 sample film with the emission spectra of the Xe lamp equipped with different filters.

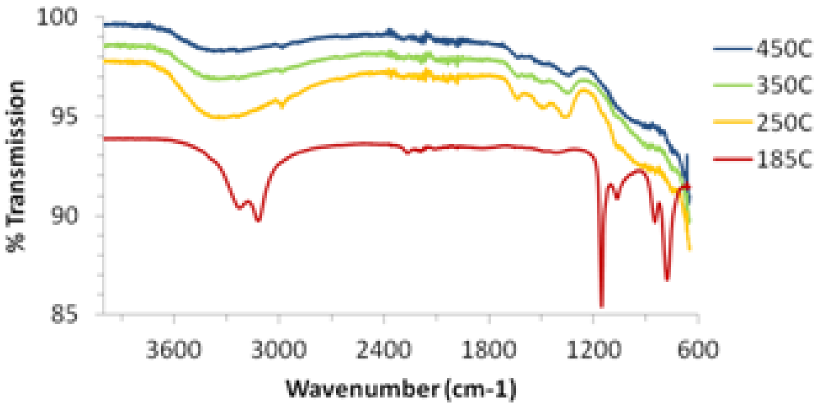

Figure S5. FT-IR-ATR spectra of $In(OH)_3$, I-250, I-350 and I-450 films used in photocatalytic reactions.

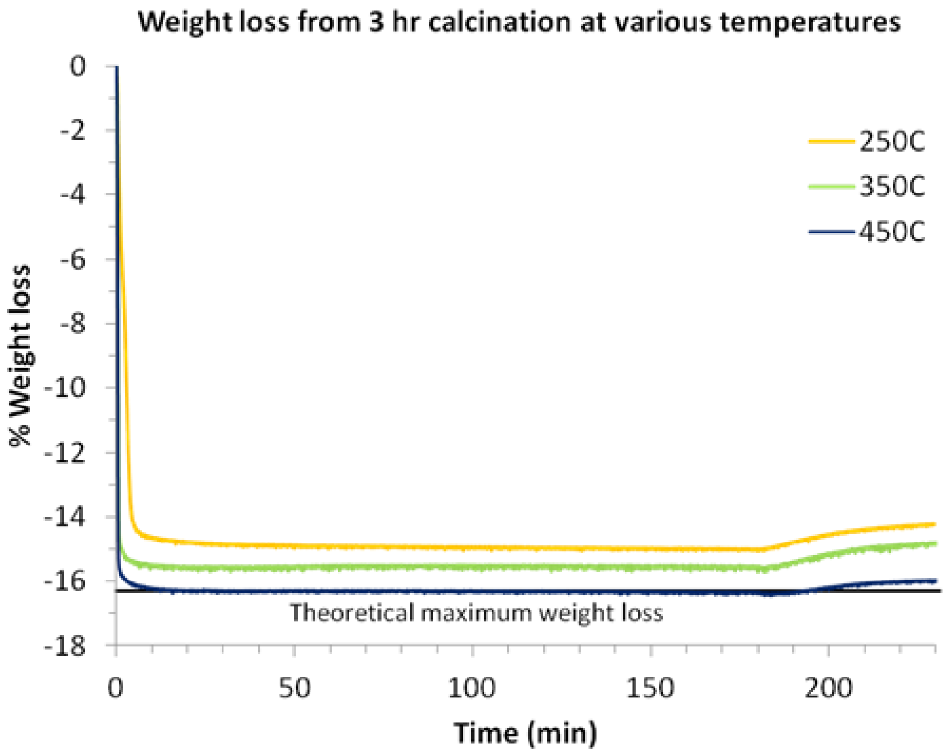

Figure S6. TGA weight loss plots mimicking the calcination process for I-250, I-350 and I-450. The black line at -16.28 % corresponds to the maximum theoretical weight loss based on stoichiometry.

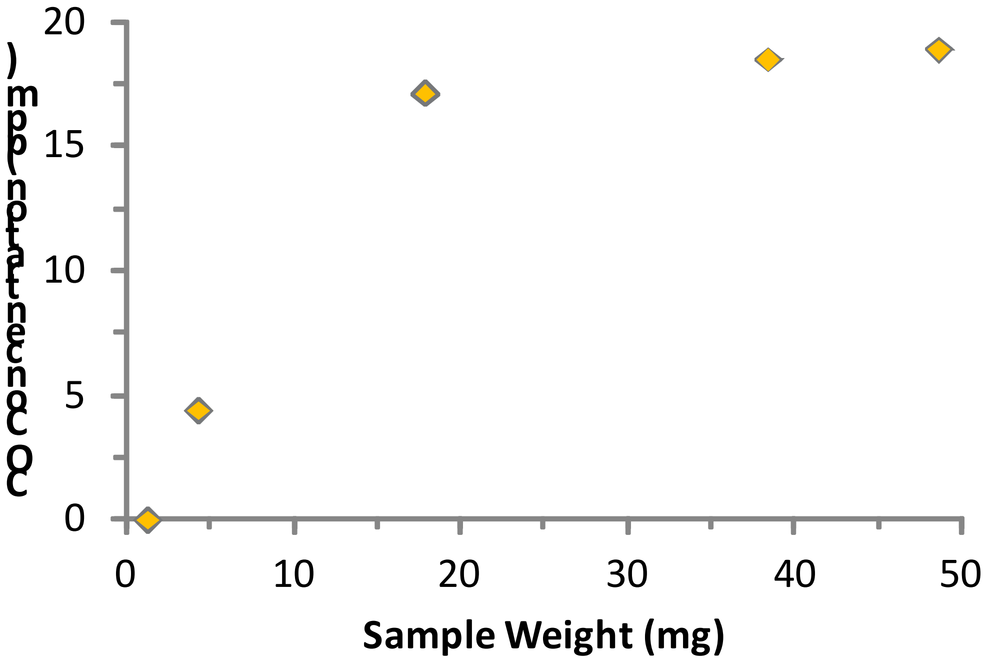

Figure S7. CO production dependence on sample loading. A series of I-250 samples with different loadings were prepared on borosilicate glass microfiber filters and tested for CO production at 150 °C under 0.8 suns of simulated solar irradiation. Based on this data a loading of 20 mg was selected for all photocatalytic measurements as it maximizes light absorption per sample weight.

a

b

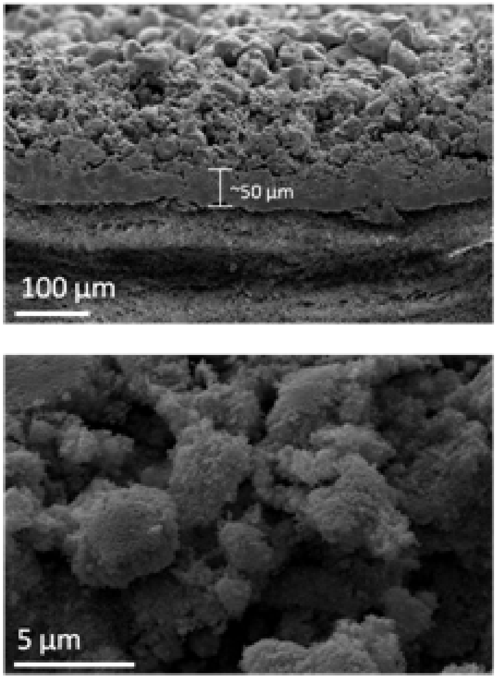

Figure S8. Cross-sectional SEM micrographs of a typical $In_2O_{3-x}(OH)_y$ film deposited on borosilicate glass microfiber filter used in catalysis testing. (a) Low magnification image showing the average film thickness is ~50 µm. (b) High magnification image show the porosity and high surface area of $In_2O_{3-x}(OH)_y$.

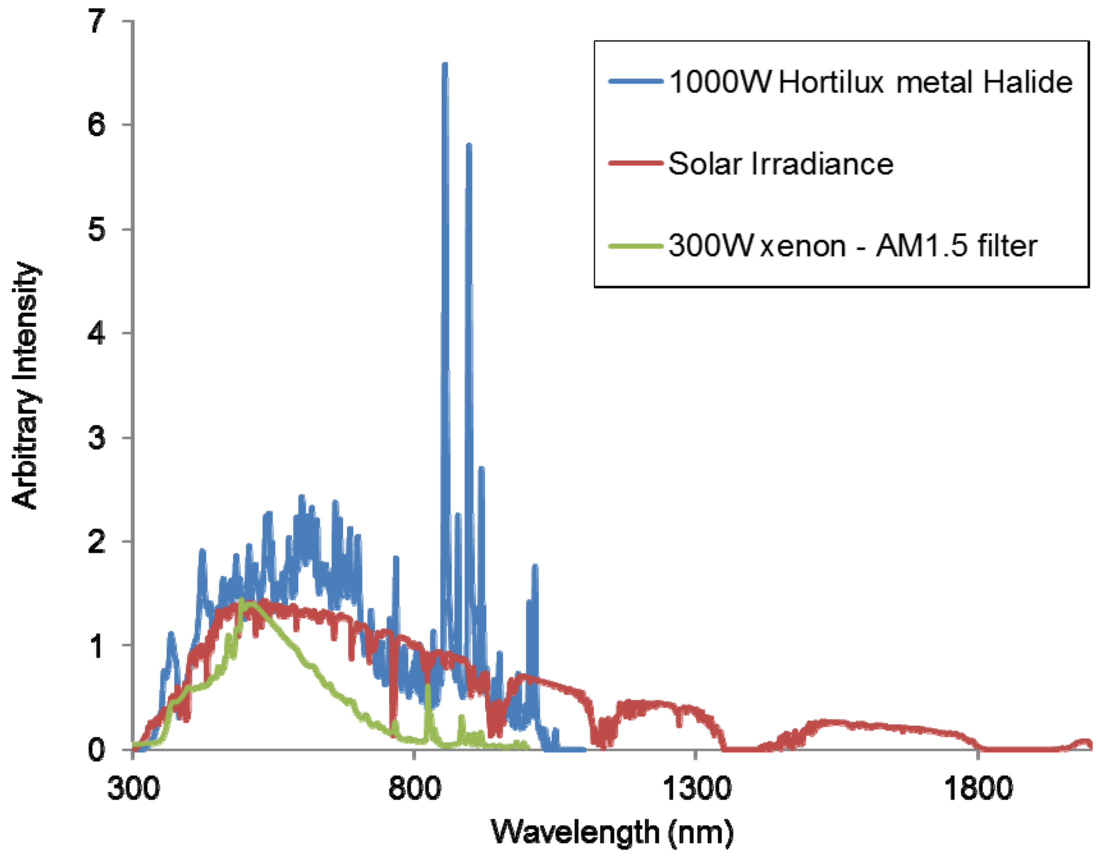

Figure S9. A comparison between the spectral distributions of the 1000 W Hortilux metal halide bulb, a 300 W Xe lamp with an AM 1.5 filter and solar irradiance.
